# Swimming Upstream: Faculty and Staff Members From Urban Middle Schools in Low-Income Communities Describe Their Experience Implementing Nutrition and Physical Activity Initiatives

**Published:** 2006-03-15

**Authors:** S Bryn Austin, Katherine W Bauer, Aarti Patel, Lisa A Prokop

**Affiliations:** Division of Adolescent and Young Adult Medicine, Children’s Hospital Boston; Division of Epidemiology and Community Health, School of Public Health, University of Minnesota, Minneapolis, Minn; Division of Adolescent and Young Adult Medicine, Children’s Hospital Boston, Boston, Mass; Division of Adolescent and Young Adult Medicine, Children’s Hospital Boston, Boston, Mass

## Abstract

**Introduction:**

Addressing childhood overweight has become a top priority in the United States. Modification of school policies and practices has been used in an attempt to address the overweight epidemic among children and adolescents. Culturally diverse urban schools in low-income communities attempting to improve nutrition and increase physical activity may face unique challenges in the school environment. A better understanding is needed about school environments and how they may affect the implementation, efficacy, and sustainability of initiatives designed to improve nutrition and physical activity.

**Methods:**

We carried out a qualitative study in five urban middle schools in low-income communities that had recently implemented Planet Health, a nutrition and physical activity intervention, to assess which aspects of the schools' physical, social, and policy environments were facilitating or impeding the implementation of health promotion initiatives. Thirty-five faculty and staff members participated. We conducted one focus group per school, with an average of seven participants per group. We analyzed focus group transcripts using the thematic analysis technique to identify key concepts, categories, and themes.

**Results:**

Teachers and staff members in our study identified many school-related environmental barriers to successful implementation of nutrition and physical activity initiatives in their schools. School personnel recommended that classroom-based nutrition interventions such as Planet Health be coordinated with school food services so that the healthy messages taught in the classroom are reinforced by the availability of healthy, culturally appropriate cafeteria food. They identified household food insufficiency and overly restrictive eligibility criteria of the federally subsidized meal program as critical barriers to healthy nutritional behaviors. They also identified weight-related teasing and bullying and unhealthy weight-control behaviors as challenges to promotion of healthy nutrition and physical activity.

**Conclusion:**

To maximize intervention efforts, researchers and practitioners must consider the effects of school environments on nutrition and physical activity initiatives.

## Introduction

Addressing childhood overweight has become a top priority in the United States. Currently, approximately 15% of children in the United States are overweight (body mass index [BMI] ≥95th percentile for age and sex) (1). Overweight among youth is associated with many health conditions, including hyperlipidemia, hypertension, and type 2 diabetes (2-7) and also contributes to substantial morbidity and mortality in adulthood (3,5-12). African American and Latino children and adolescents are at greater risk for overweight than white children and adolescents (1,8,13-15). In 1998, a national survey of children aged 4 to 12 years found that approximately 21% of African American and Latino children were overweight, compared with 12% of white children, and 35% of African American and Latino children were at risk for overweight (85th percentile to <95th percentile BMI for age and sex), compared with 20% of white children (14).

Overweight children are more likely to be targeted by bullies than children who are not overweight (16,17). Weight-related teasing and harassment increase the risk of unhealthy weight-control behaviors, such as self-induced vomiting and misuse of laxatives and diet pills, and eating disorders among adolescents (18,19). By high school, 10% of Latina girls, 6% of African American girls, and 9% of white girls report having induced vomiting or using laxatives in the previous month to lose or maintain weight (20).

Modifying school policies and practices to improve nutrition and increase physical activity has become a focus in the effort to address the overweight epidemic among children and adolescents (21-24). Students consume a substantial portion of their daily calories at school, and most of their weekday physical activity occurs during or immediately before and after school (25-27). Interventions designed to modify school policies, procedures, and curricula have improved student nutrition, increased physical activity, decreased television viewing (28-32), and reduced overweight (31,32). An understanding of the way the school environment affects the implementation, efficacy, and sustainability of an intervention is needed to maximize the impact of school-based health initiatives (24,26,27,29,33). Interventions implemented in schools in low-income urban communities may have unique challenges related to their limited resources. Identifying and addressing these challenges while planning and implementing school-based nutrition and physical activity initiatives are essential.

Planet Health is a nutrition and physical activity intervention for middle schools that has effectively reduced overweight (32). The intervention consists of lesson plans that are integrated into the school curriculum during 2 school years. The lesson plans emphasize making nutritious food choices, increasing physical activity, and decreasing time spent watching television (32,34).

Wiecha et al examined the fidelity of Planet Health implementation and the feasibility and sustainability of the curriculum in six urban public middle schools serving low-income communities (35). Teachers participating in the study said that Planet Health was acceptable and feasible, and most reported a high level of perceived competence in teaching the health concepts outlined in the program. Limitations in intervention implementation noted by Wiecha et al included varying from the original intervention design and incomplete implementation of the lesson plans. Wiecha et al also noted that teachers and staff members identified the school meal program, vending machines, and certain school system attributes as environmental challenges to intervention goals (35).

Building on the Wiecha evaluation, we carried out a second study in five of the same six schools. We used qualitative methods to assess which aspects of the schools' physical, social, and policy environments facilitate or impede the implementation of Planet Health and other initiatives designed to promote healthy nutrition and physical activity in urban middle schools in low-income communities.

## Methods

### Study sample and research design

We obtained the study sample from the teaching and administrative staff of five of the six public middle schools in Boston that were included in the study by Wiecha et al (35). One of the six schools chose not to take part in our study. All of the schools had implemented Planet Health during the previous 1 or 2 school years. The average enrollment of the five middle schools was approximately 600 students, and all included grades 6 through 8; one school included prekindergarten through grade 5. At all five schools, more than 75% of the student body was African American or Latino. English was not the first language for at least 20% of the students, and between 80% and 90% of students were eligible for free or reduced-price lunch.

We invited faculty and staff members who had been involved in the Planet Health implementation to participate in a focus group. We conducted one focus group lasting approximately 1 hour at each school during the school day and provided lunch as an incentive. Thirty-five participants attended the focus groups, with an average of seven participants per group. The groups were led by a clinical social worker experienced in focus group moderation. Participants included school administrators and teachers of math, social sciences, language arts, physical education (PE), special education, and health. The focus groups were tape-recorded, and the tapes were transcribed verbatim. Data were collected in 2001 and 2002, and participants provided written informed consent at the time of the focus groups. The institutional review board of Children's Hospital Boston approved the study.

A semistructured focus group moderator's guide was developed based on current research on the influences of physical, social, and policy environments on adolescent nutrition and physical activity behavior (24,33,36). The ecological model, which served as the theoretical basis for the guide, proposes that individual health behaviors are influenced by the environment on many levels, with influences including interpersonal relationships and organizational and institutional factors (37,38). Topics addressed in the focus groups included access to healthy and unhealthy food in school, opportunities for physical activity, the relationships between the Planet Health intervention and the school environment, school policies on weight-related harassment, and student weight-control practices.

### Data coding and analysis

Transcripts of focus group sessions were analyzed by the research team using the thematic analysis technique (39,40); key concepts, categories, and themes were identified. During a series of meetings, the research team discussed preliminary findings and grouped emerging themes to develop an analytic framework of relationships between the school environment and nutrition and physical activity initiatives. The team used the resulting analytic framework to define organizational codes to facilitate qualitative analysis using the software package NUD*IST Vivo (Qualitative Solutions and Research Pty Ltd, Doncaster, Australia). All transcripts were coded independently by two members of the research team (K.W.B. and A.P.). In the analysis framework, we subdivided the school environment factors into three health domains: nutrition opportunities, physical activity opportunities, and weight-related teasing and weight-control practices.

## Results

### Nutrition opportunities

None of the five schools participating in the study had full kitchens; they had minimal equipment such as sinks and warming ovens. Meals were prepared at the school district's central kitchen and then heated in each school's kitchen, giving schools little control over food offered in the cafeteria. Focus group participants described the effects they believed food service policies and practices had on student food choices, saying that their schools did not provide enough healthy choices for students. One teacher explained:

We tell them to eat well, and we serve them french fries every day, and I know that they're baked, I know they're not actually fried, but, come on. Everything's in cellophane. [We teach them] don't eat prepackaged foods, they're not as nutritious as the fresh cooked foods, and everything they get they have to unwrap the little cellophane wrapper. It's disgusting.

Two staff members at another school made similar observations, expressing their frustration with the ways that food service policies and practices undermined nutrition education efforts:

*Participant 1:* One thing that I've noticed is that a lot of kids, like probably 60% or 70%, eat chips for lunch. And on the snack cart that they have — they have a regular lunch that kids can get free or buy — but on the cart they don't have any nutritious things, just ice cream and all the junk stuff.

*Participant 2:* There is a health class, but you see contradictions. The kids come out of health class, and they go down to the cafeteria — the system undermines [the health messages]. It's swimming upstream.

Some teachers reported that during the school year, the students began to notice the contradictions between nutritional behaviors encouraged by teachers and the options provided in the school cafeterias. One teacher said the following about the students:

They talk about the cookies and the chips and some of the food [offered in the cafeteria]. And they go, "Well, if all these things are so bad for us, you know, and they're supposed to serve us healthy lunches and balanced lunches, why are they serving what they're serving . . . all this stuff that's going to kill our arteries, and knock us out?"

A theme that emerged from all the focus groups was that Planet Health and other nutrition education efforts needed to be incorporated into the school food services. One staff member explained, "I think the easiest thing to do really would be align Planet Health with the cafeteria. It would really make a greater impact. And then what we do is piggyback off of that." At another school, a teacher suggested that Planet Health "would have a lot more impact if they [the students] could see the lessons reflected in the food they are served."

Many faculty and staff members said that they believed the widespread problem of overweight, especially among African American and Latino children and children from low-income families, made it imperative to serve healthier food in their schools. They believed that it was the responsibility of the school to compensate for the lack of nutritious food in many of the students' homes. Faculty and staff members explained that some of their students were living in households without enough food because the families did not always have enough money for groceries. They said that these children would often arrive at school having eaten little at home, or they would bring to school inexpensive but nonnutritious foods such as soda, potato chips, and candy that they had purchased at convenience stores close to the school. A teacher described one student who found it nearly impossible to adhere to the dietary restrictions recommended by her doctor because of a lack of food at home and a lack of nutritious offerings at school: "I have another kid, in eighth grade, who won't eat lunch because the lunch has too much sodium, too much this, all these things in it that she can't have. . . . And she should bring a lunch from home, but there's nothing at home to bring."

Teachers said that they had students who did not qualify for the subsidized meal program according to the current federal eligibility restrictions but nevertheless could not afford to buy lunch at school. Teachers stated that students' fears about seeming poor or hungry made them reluctant to ask for or accept money or food offered by school staff. Faculty and staff members mentioned that some students who did qualify for free or reduced-price meals felt self-conscious as they passed through the lunch line because they knew that peers near them would find out that they were receiving subsidized meals. One teacher explained:

Two years ago, I realized that half my class was not eligible for the free lunch. And they were not bringing any money. I have offered to pay for their lunch, and they say, "I'm not hungry," because it's embarrassing. And then, some of them even say things such as "I don't want to get fat" just to pretend that they're on a diet, because they really cannot afford it, yet they're not eligible for it.

Some participants described instances in which school cafeterias had too little food to serve all the students who wanted to purchase lunch. One teacher described several occasions on which she believed her school cafeteria did not have enough food to last through all lunch periods in a day or until the end of the week.

Many participants commented on the influence that cultural background and teasing by peers had on student food choices. Some believed that while at school, students whose families had recently immigrated to the United States were not eating some of the healthy ethnic food they typically ate at home either because the food was not available in the cafeteria or because they were teased for bringing the food from home. One teacher offered the following perspective:

We serve a high immigrant population. Most of the parents — the vast majority of the parents — are immigrants. And kids first come to the United States, they may have ate healthy in their homeland. They come to the United States, it's not cool to play soccer, it's not cool to do other things, eat fresh fruit. . . . The immigrant parents are very aware of health but their offspring are coming at it just the other way and saying, "I want to be an American. Americans eat chips, hot dogs." So it's, you know, it must be very hard for parents to offset this.

### Physical activity opportunities

Participants identified many school-related barriers to student participation in physical activity. Factors included academic and medical examination requirements for participation in athletic teams, infrequent PE classes and after-school physical activities, and a lack of outdoor playing space. For example, various extracurricular sports teams were available at each of the schools; however, students were required to maintain a C average to qualify for participation. In addition, schools required students to get a medical examination before joining a sports team, which was financially difficult for low-income families and prevented some students from participating.

PE class scheduling was inconsistent in some of the schools. Some students had daily PE class during the first semester but no classes at all during the second semester. In other schools, students in certain grades were not offered PE classes during certain times of the year; in one school, an entire grade did not have PE class for the whole school year because of a scheduling error. Many of the schools did not have outdoor grass fields where students could play sports. Some schools improvised by having students play on a parking lot near the school campus. The lack of outside play space limited the types of activities offered in PE class and the number of sports teams that the schools could support.

Even when PE classes were offered, staff members explained that several barriers kept many students, especially girls and overweight students, from participating fully in class. They said many girls were uncomfortable playing sports with boys and thought they did not have enough privacy in locker rooms. In PE classes in which girls outnumbered boys, girls seemed to enjoy participating in class. One teacher described the way her class of primarily girls compared with other classes: "They seem to love gym. I would always see them carrying their gym clothes and deodorant and all these little things. Whereas other kids, I go pick them up from gym, and they wouldn't have anything; they wouldn't have gym clothes." Many staff members commented that students were reluctant to change in front of each other in the locker rooms. One teacher explained, "There's no shower, and there's no privacy in the changing area. For someone who's self-conscious, especially one who might be on the obese side, there's no [private] bathing, shower facilities."

Teachers pointed out that some students who disliked PE classes and found the PE class environment unsupportive enjoyed physical activity in other settings. A teacher at one school recalled her experience coaching an after-school Special Olympics program:

There are several students that really, really hate gym that participate [in the Special Olympics program]. I do Special Olympics track, in the spring, it's an after-school activity. And all the students with [disabilities] have partners that are just the regular kids that help them. And a lot — I mean, I want to say almost all of my partners that run after school, do the routines after school, work out with their special partners — are the same ones that hate gym. So it's not the physical activity that they hate. . . . They'll run when they're helping out a girl with Down syndrome, but they don't want to do it in front of the eighth-grade boys.

### Weight-related teasing and concerns

Participants observed that weight-related teasing is very common in their middle schools, and one teacher even described it as "brutal." Participants thought that weight-related bullying was not taken seriously enough by administrators and reported that their schools did not have explicit policies on preventing or managing incidents. They pointed out that teachers were expected to manage weight-related teasing and harassment on their own, whereas sexual harassment incidents received immediate attention from the administration. A staff member spoke about how changes needed to occur on the school level to decrease weight-related and other bullying in schools:

It's not only your class. It's the school thing, and it involves a different message. And maybe we should embrace that. We can embrace that we want all of our kids to be literate. We also want to be bully-free. . . . So I think that's a campaign that we need to do as the whole school.

Many teachers expressed concern about how their students' weight-control behaviors seemed to negatively affect their eating patterns. They noted that some students were not eating balanced meals because of weight concerns, a poor body image, and peer pressure to diet. One faculty member explained: "There are a lot of issues, I think especially with the girls, with eating. We have [everything from] girls that won't eat the school lunch, don't eat anything — who are very thin — to girls watching their weight."

At one school, staff members discussed the way a student who they believed had an eating disorder had been affecting other students. The girl had been eating very little, and the other students in her class, especially the girls, were aware of her unhealthy weight-control behaviors. One teacher explained, "She was influential because she told the others, 'This is how you're supposed to look.' The students . . . they wouldn't even eat lunch last year. None of them would eat lunch, none of them." The teachers said they explained to the other girls that the student was not an appropriate role model for proper eating habits, but the peer pressure to participate in unhealthy weight-control behaviors was strong. Several staff members said that they wanted training on recognizing and addressing eating disorders in addition to the training they received on promoting healthy nutrition and physical activity through the Planet Health intervention.

## Discussion

Determining successful ways to work with schools to improve the physical activity and nutrition opportunities for students is essential for reducing the epidemic of overweight and its related health consequences. All of the schools participating in our study had demonstrated a commitment to addressing student nutrition and physical activity issues by adopting the Planet Health intervention schoolwide. Yet even in these schools, faculty and staff members identified myriad environmental barriers that undermined efforts to promote healthy nutrition and physical activity.

In a previous study conducted in suburban middle schools by members of our research team, we found many barriers to healthy nutrition and physical activity in schools, including school meals that students found unappealing, easy access to nonnutritious foods in the schools, and teasing and harassment of students during PE classes (33). In our current study, which was conducted in urban schools with fewer resources than their suburban counterparts, we found that some of the barriers were the same but also discovered additional challenges related to inadequate school funding for nutrition and physical activity programs and economic hardships faced by families. Our study results are consistent with those of Wiecha et al (35), who noted that school staff members identified school meal programs and other school-related characteristics as barriers to nutrition and physical activity promotion.

Participants in our study emphasized that the lack of eligibility for subsidized meals was a critical challenge to nutrition promotion initiatives in schools in low-income communities. Faculty and staff members reported that many students did not qualify for federal assistance through the subsidized meal program but still could not afford the school lunch. Increasing access to healthy meals provided at school is especially important for children living in food-insufficient households (households in which family members have limited access to nutritionally adequate or safe foods) (41,42). Immigrants and children from low-income families are at greatest risk for food insufficiency (42). In 2000, approximately 14 million children younger than 18 years in the United States were living in food-insufficient households (43). Food insufficiency has been associated with a range of negative physical and psychosocial consequences, including delayed academic and emotional development and possibly an increased risk for overweight (41,43). To address food insufficiency among students, the federal Child Nutrition and Women, Infants, and Children (WIC) Reauthorization Act of 2004 included a plan, but not funding, for a pilot project to eliminate the reduced-price lunch category and expand the proportion of low-income students eligible for fully subsidized meals. Hundreds of child health organization and school boards are advocating for Congress to authorize funding for the pilot program in five states so that the initiative's efficacy can be evaluated (44) (C. Schuchart, oral communication, November 2005.) Policy changes ensuring that all children have access to nutritionally balanced meals at school could reduce the risks associated with household food insufficiency and improve the nutritional status of U.S. children.

Teachers and staff members made several recommendations for addressing barriers that undermined Planet Health and other nutrition and physical activity initiatives in their schools:

- Teachers and staff members strongly recommended that nutrition interventions such as Planet Health also be coordinated with school food services so that healthy classroom messages are reinforced by healthy food in the cafeteria that is accessible, affordable, and culturally appropriate.
- Teachers and staff members recommended offering more physical activity alternatives to standard PE classes to appeal to more students, especially girls and overweight students, and developing alternative activities to compensate for the inadequate outdoor play spaces.
- Weight-related teasing and bullying was perceived as a pervasive problem, so teachers and staff members recommended making more coordinated schoolwide efforts to prevent bullying and obtaining support from school administrators.
- Teachers and staff members asked for more training on eating disorders so that they could identify students who needed treatment and learn ways to handle students who may negatively influence the weight-control behaviors of other students.

Limitations of the study include the use of self-reported data without supplemental objective data sources. In addition, certain important factors in the schools' physical, social, and policy environments may have not been addressed because participants were hesitant to speak about them. Participants in the focus groups were colleagues, which may have led them to respond less candidly. Lastly, the perspectives of stakeholders in school communities who did not participate in the study, such as food services staff members, students, and parents, may vary from those of the people who attended the focus group sessions.

Teachers and staff members in our study recognized the need for school-based nutrition and physical activity programs such as Planet Health, but they identified many environmental barriers to complete and successful implementation of interventions in their culturally diverse urban schools in low-income communities. To maximize intervention efforts, researchers and practitioners must pay careful attention to the influence of school environments on nutrition and physical activity initiatives.

## References

[1] Ogden CL, Flegal KM, Carroll MD, Johnson CL (2002). Prevalence and trends in overweight among U.S. children and adolescents, 1999-2000. JAMA.

[2] American Diabetes Association (2000). Type 2 diabetes in children and adolescents. Pediatrics.

[3] Drake AJ, Smith A, Betts PR, Crowne EC, Shield JP (2002). Type 2 diabetes in obese white children. Arch Dis Child.

[4] Goran MI, Ball GD, Cruz ML (2000). Obesity and risk of type 2 diabetes and cardiovascular disease in children and adolescents. J Clin Endocrinol Metab.

[5] Freedman DS, Dietz WH, Srinivasan SR, Berenson GS (1999). The relation of overweight to cardiovascular risk factors among children and adolescents: the Bogalusa Heart Study. Pediatrics.

[6] Morrison JA, Barton BA, Biro FM, Daniels SR, Sprecher DL (1999). Overweight, fat patterning, and cardiovascular disease risk factors in black and white boys. J Pediatr.

[7] Morrison JA, Sprecher DL, Barton BA, Waclawiw MA, Daniels SR (1999). Overweight, fat patterning, and cardiovascular disease risk factors in black and white girls: The National Heart, Lung, and Blood Institute Growth and Health Study. J Pediatr.

[8] Wang Y (2001). Cross-national comparison of childhood obesity: the epidemic and the relationship between obesity and socioeconomic status. Int J Epidemiol.

[9] Power C, Lake JK, Cole TJ (1997). Measurement and long-term health risks of child and adolescent fatness. Int J Obes Relat Metab Disord.

[10] Bray GA, Bouchard C, James WPT (1998). Handbook of obesity.

[11] Must A, Strauss RS (1999). Risks and consequences of childhood and adolescent obesity. Int J Obes Relat Metab Disord.

[12] Fagot-Campagna A, Pettitt DJ, Engelgau MM, Burrows NR, Geiss LS, Valdez R (2000). Type 2 diabetes among North American children and adolescents: an epidemiologic review and a public health perspective. J Pediatr.

[13] Mirza NM, Kadow K, Palmer M, Solano H, Rosche C, Yanovski JA (2004). Prevalence of overweight among inner city Hispanic-American children and adolescents. Obes Res.

[14] Strauss RS, Pollack HA (2001). Epidemic increase in childhood overweight, 1986–1998. JAMA.

[15] Winkleby MA, Robinson TN, Sundquist J, Kraemer HC (1999). Ethnic variation in cardiovascular disease risk factors among children and young adults: findings from the Third National Health and Nutrition Examination Survey, 1988–1994. JAMA.

[16] Neumark-Sztainer D, Falkner N, Story M, Perry C, Hannan PJ, Mulert S (2002). Weight-teasing among adolescents: correlations with weight status and disordered eating behaviors. Int J Obes Relat Metab Disord.

[17] Janssen I, Craig WM, Boyce WF, Pickett W (2004). Associations between overweight and obesity with bullying behaviors in school-aged children. Pediatrics.

[18] Lieberman M, Gauvin L, Bukowski WM, White DR (2001). Interpersonal influence and disordered eating behaviors in adolescent girls: the role of peer modeling, social reinforcement, and body-related teasing. Eat Behav.

[19] The McKnight Investigators (2003). Risk factors for the onset of eating disorders in adolescent girls: results of the McKnight Longitudinal Risk Factor Study. Am J Psychiatry.

[20] (2004). Centers for Disease Control and Prevention. Youth Risk Behavior Surveillance — United States, 2003. MMWR Morb Mortal Wkly Rep.

[21] (2001). Increasing physical activity. A report on recommendations of the Task Force on Community Preventive Services. MMWR Recomm Rep.

[22] Centers for Disease Control and Prevention (1996). Guidelines for school health programs to promote lifelong healthy eating. MMWR Recomm Rep.

[23] Perry CL (1999). Creating health behavior change: how to develop community-wide programs for youth.

[24] Neumark-Sztainer D (1999). The social environments of adolescents: associations between socioenvironmental factors and health behaviors during adolescence. Adolesc Med.

[25] Davison KK, Birch LL (2001). Childhood overweight: a contextual model and recommendations for future research. Obes Rev.

[26] Kubik MY, Lytle LA, Hannan PJ, Perry CL, Story M (2003). The association of the school food environment with dietary behaviors of young adolescents. Am J Public Health.

[27] French SA, Story M, Fulkerson JA, Gerlach AF (2003). Food environment in secondary schools: a la carte, vending machines, and food policies and practices. Am J Public Health.

[28] Luepker RV, Perry CL, McKinlay SM, Nader PR, Parcel GS, Stone EJ (1996). Outcomes of a field trial to improve children's dietary patterns and physical activity: the Child and Adolescent Trial for Cardiovascular Health (CATCH). JAMA.

[29] Sallis JF, McKenzie TL, Conway TL, Elder JP, Prochaska JJ, Brown M (2003). Environmental interventions for eating and physical activity: a randomized controlled trial in middle schools. Am J Prev Med.

[30] Jamner MS, Spruijt-Metz D, Bassin S, Cooper DM (2004). A controlled evaluation of a school-based intervention to promote physical activity among sedentary adolescent females: Project FAB. J Adolesc Health.

[31] Robinson TN (1999). Reducing children's television viewing to prevent obesity, a randomized controlled trial. JAMA.

[32] Gortmaker SL, Peterson K, Wiecha J, Sobol AM, Dixit S, Fox MK (1999). Reducing obesity via a school-based interdisciplinary intervention among youth: Planet Health. Arch Pediatr Adolesc Med.

[33] Bauer KW, Yang YW, Austin SB (2004). "How can we stay healthy when you're throwing all this in front of us?" Findings from focus groups and interviews in middle schools on environmental influences on nutrition and physical activity. Health Educ Behav.

[34] Carter J, Wiecha J, Peterson K, Gortmaker SL (2001). Planet Health: an interdisciplinary curriculum for teaching middle school nutrition and physical activity. Human Kinetics.

[35] Wiecha JL, El Ayadi AM, Fuemmeler BF, Carter JE, Handler S, Johnson S (2004). Diffusion of an integrated health education program in an urban school system: Planet Health. J Pediatr Psychol.

[36] Neumark-Sztainer D, Story M, Perry C, Casey MA (1999). Factors influencing food choices of adolescents: findings from focus-group discussions with adolescents. J Am Diet Assoc.

[37] Bronfenbrenner U (1979). The ecology of human development: experiments by nature and design.

[38] Stokols D (1996). Translating social ecological theory into guidelines for community health promotion. Am J Health Promot.

[39] Miles MB, Huberman AM (1994). Qualitative data analysis: an expanded sourcebook.

[40] Rice P, Ezzy D (1999). Qualitative research methods. A health focus.

[41] Alaimo K, Olson C, Frongillo EA, Briefel RR (2001). Food insufficiency, family income, and health in U.S. preschool and school-aged children. Am J Public Health.

[42] Mitka M (2002). Not enough food (instead of too much) is also a problem in the United States. JAMA.

[43] Alaimo K, Olson C, Frongillo EA (2001). Low family income and food insufficiency in relation to overweight in U.S. children: is there a paradox?. Arch Pediatr Adolesc Med.

[44] Johnson K (2005). Congressional testimony, School Nutrition Association. Congressional Quarterly.

